# Supplementary material for: From sequence to enzyme mechanism using multi-label machine learning
Source: BMC Bioinformatics. 2014 May 19;15:150. doi: 10.1186/1471-2105-15-150 (PMC4229970; doi:10.1186/1471-2105-15-150)
Supplement: Additional file 2 — Java code of ml2db. Additional file ml2db_code.tar.gz contains the Java source code to run the multi-label machine learning experiments and save the results to database. The code’s Javadoc is included. [file 1471-2105-15-150-S2.zip › additional file 2/ml2db/ml2db/doc/test/ml2db/mulan/attributesfilter/AttFiltArffTest.html]

AttFiltArffTest


JavaScript is disabled on your browser.


- Overview
- Package
- Class
- Use
- Tree
- Deprecated
- Index
- Help

- Prev Class
- Next Class

- Frames
- No Frames

- All Classes

- Summary:
- Nested |
- Field |
- Constr |
- Method

- Detail:
- Field |
- Constr |
- Method


test.ml2db.mulan.attributesfilter

## Class AttFiltArffTest

- java.lang.Object
- - junit.framework.Assert
  - - junit.framework.TestCase
    - - test.ml2db.mulan.attributesfilter.AttFiltArffTest

- All Implemented Interfaces:
  :   junit.framework.Test

  ---

    

  ```
  public class AttFiltArffTest
  extends junit.framework.TestCase
  ```

- - ### Field Summary

    Fields

    | Modifier and Type | Field and Description |
    | --- | --- |
    | `static int` | `TEST_ROLE` |
    | `static int` | `TRAIN_ROLE` |
  - ### Constructor Summary

    Constructors

    Constructor and Description

    `AttFiltArffTest()`
  - ### Method Summary

    Methods

    | Modifier and Type | Method and Description |
    | --- | --- |
    | `static java.lang.String` | `getCorrectClassAttributes()` |
    | `static java.lang.String` | `getCorrectFeatureAttributes()` |
    | `static int` | `getFileRole(java.lang.String fileName)` Returns the role ("train" or "test") of the file for a potential train/test evaluation. |
    | `static int[]` | `getNewFilteredArffs()` Get the new, filtered, train and test arff files |
    | `static void` | `testANewFilteredFileContent(int role, int newId, java.lang.String[] content)` |
    | `void` | `testArffName()` |
    | `void` | `testNewTestsetFile()` |
    | `void` | `testNewTrainsetFile()` |

    - ### Methods inherited from class junit.framework.TestCase

      `countTestCases, getName, run, run, runBare, setName, toString`
    - ### Methods inherited from class junit.framework.Assert

      `assertEquals, assertEquals, assertEquals, assertEquals, assertEquals, assertEquals, assertEquals, assertEquals, assertEquals, assertEquals, assertEquals, assertEquals, assertEquals, assertEquals, assertEquals, assertEquals, assertEquals, assertEquals, assertEquals, assertEquals, assertFalse, assertFalse, assertNotNull, assertNotNull, assertNotSame, assertNotSame, assertNull, assertNull, assertSame, assertSame, assertTrue, assertTrue, fail, fail, failNotEquals, failNotSame, failSame, format`
    - ### Methods inherited from class java.lang.Object

      `equals, getClass, hashCode, notify, notifyAll, wait, wait, wait`

- - ### Field Detail


    - #### TRAIN\_ROLE

      ```
      public static final int TRAIN_ROLE
      ```

      See Also:
      :   Constant Field Values


    - #### TEST\_ROLE

      ```
      public static final int TEST_ROLE
      ```

      See Also:
      :   Constant Field Values
  - ### Constructor Detail


    - #### AttFiltArffTest

      ```
      public AttFiltArffTest()
      ```
  - ### Method Detail


    - #### testArffName

      ```
      public void testArffName()
      ```


    - #### testNewTestsetFile

      ```
      public void testNewTestsetFile()
      ```


    - #### testNewTrainsetFile

      ```
      public void testNewTrainsetFile()
      ```


    - #### getCorrectClassAttributes

      ```
      public static java.lang.String getCorrectClassAttributes()
      ```


    - #### getCorrectFeatureAttributes

      ```
      public static java.lang.String getCorrectFeatureAttributes()
      ```


    - #### getFileRole

      ```
      public static int getFileRole(java.lang.String fileName)
      ```

      Returns the role ("train" or "test") of the file for a potential
      train/test evaluation. A filtered train file has the same id for the
      filtered file \*and\* for the attributes. A filtered test file has
      different id for the filtered file and the attributes (because the
      attributes come from the old train file).

      Parameters:
      :   `fileName` - a filtered arff file name

      Returns:
      :   0 if a train file, 1 if a test file


    - #### getNewFilteredArffs

      ```
      public static int[] getNewFilteredArffs()
      ```

      Get the new, filtered, train and test arff files

      Returns:
      :   an array where the first element is the new train arff and the
          second element is the new test arff


    - #### testANewFilteredFileContent

      ```
      public static void testANewFilteredFileContent(int role,
                                     int newId,
                                     java.lang.String[] content)
      ```


- Overview
- Package
- Class
- Use
- Tree
- Deprecated
- Index
- Help

- Prev Class
- Next Class

- Frames
- No Frames

- All Classes

- Summary:
- Nested |
- Field |
- Constr |
- Method

- Detail:
- Field |
- Constr |
- Method
